# Interactions between β Subunits of the KCNMB Family and Slo3: β4 Selectively Modulates Slo3 Expression and Function

**DOI:** 10.1371/journal.pone.0006135

**Published:** 2009-07-03

**Authors:** Cheng-Tao Yang, Xu-Hui Zeng, Xiao-Ming Xia, Christopher J. Lingle

**Affiliations:** Department of Anesthesiology, Washington University School of Medicine, St. Louis, Missouri, United States of America; Harvard Medical School, United States of America

## Abstract

**Background:**

The pH and voltage-regulated Slo3 K^+^ channel, a homologue of the Ca^2+^- and voltage-regulated Slo1 K^+^ channel, is thought to be primarily expressed in sperm, but the properties of Slo3 studied in heterologous systems differ somewhat from the native sperm KSper pH-regulated current. There is the possibility that critical partners that regulate Slo3 function remain unidentified. The extensive amino acid identity between Slo3 and Slo1 suggests that auxiliary β subunits regulating Slo1 channels might coassemble with and modulate Slo3 channels. Four distinct β subunits composing the KCNMB family are known to regulate the function and expression of Slo1 Channels.

**Methodology/Principal Findings:**

To examine the ability of the KCNMB family of auxiliary β subunits to regulate Slo3 function, we co-expressed Slo3 and each β subunit in heterologous expression systems and investigated the functional consequences by electrophysiological and biochemical analyses. The β4 subunit produced an 8–10 fold enhancement of Slo3 current expression in *Xenopus* oocytes and a similar enhancement of Slo3 surface expression as monitored by YFP-tagged Slo3 or biotin labeled Slo3. Neither β1, β2, nor β3 mimicked the ability of β4 to increase surface expression, although biochemical tests suggested that all four β subunits are competent to coassemble with Slo3. Fluorescence microscopy from β4 KO mice, in which an eGFP tag replaced the deleted exon, revealed that β4 gene promoter is active in spermatocytes. Furthermore, quantitative RT-PCR demonstrated that β4 and Slo3 exhibit comparable mRNA abundance in both testes and sperm.

**Conclusions/Significance:**

These results argue that, for native mouse Slo3 channels, the β4 subunit must be considered as a potential interaction partner and, furthermore, that KCNMB subunits may have functions unrelated to regulation of the Slo1 α subunit.

## Introduction

An essential step in mammalian fertilization is an enhanced sperm motility termed hyperactivation associated with an elevation in cysotolic Ca^2+^ [1], [2]. This process is initiated by an increase in cytosolic pH, which in mouse spermatocytes activates Ca^2+^-permeable CatSper channels [3] and, in concert, a pH-regulated K^+^ current termed KSper [4]. The identity of the molecular substrate for KSper remains uncertain, although the primary candidate is the pH-regulated Slo3 K^+^ channel, expressed perhaps exclusively in spermatocytes [5]. However, differences in the voltage-dependence of gating between the native KSper current and heterologously expressed Slo3 current raise some question as to their equivalence. One possibility is that Slo3 channel complex in native cells involves other components that help define the properties of the native KSper current.

Slo3 shares extensive amino acid identity with Slo1, which encodes the pore-forming α subunit of the BK-type Ca^2+^- and voltage-activated K^+^ channel [5]–[7]. Membrane-residing domains of Slo1 and Slo3 share approximately 56% identity [5], while the cytosolic domains of Slo1 and Slo3, which confer ligand dependence [8], share about 39% amino acid identity [5]. BK channels in native cells often contain auxiliary β subunits encoded by the KCNMB gene family [9]–[12], that determine a variety of functional properties of BK channels including apparent Ca^2+^-dependence, gating kinetics, inactivation behavior, and pharmacology [11], [13]–[15]. Furthermore, some β subunits influence trafficking of BK channels [16], [17]. Despite the clear role that KCNMB subunits play in defining properties of BK channels, there are tissues that may abundantly express particular KCNMB subunits, but may lack significant Slo1 α subunit expression [18, www.ncbi.nlm.nih.gov/UniGene]. This raises the possibility that KCNMB subunits may have roles unrelated to BK channels. Here, we have examined the ability of the so-called BK β subunits to influence Slo3 gating properties and membrane expression. We also tested the possibility that a β subunit may be a candidate auxiliary subunit of Slo3 subunits in mouse sperm.

## Materials and Methods

### Animals and Oocyte Culture

All procedures involving mice and *Xenopus laevis* were conducted in accordance to Guidelines for the Care and Use of Research Animals established by The Animal Studies Committee of Washington University. The procedures for preparation and injection of stage IV *Xenopus laevis* oocytes were as previously described [19]. The concentration of mSlo3 cRNA was around 1 µg/µl with no dilution. Oocytes were typically used 3–7 days after injection. For comparisons of the relative expression levels of Slo3 with and without a particular β subunit, sets of oocytes were injected with either Slo3 alone or Slo3+β subunit. The absolute amount of injected Slo3 was kept constant for all such injections (∼10 ng), while β subunit cRNA was injected in half the oocytes at a ratio of 1∶2 Slo3/β by weight.

### Chimeric constructs and mutations

The mSlo3 construct [5] (obtained from L. Salkoff, Washington Univ. Sch. Med., St. Louis) was modified as previously described [8]. The accession number for Slo3 is AF039213. For all β subunits, human forms were typically used (Accession numbers: NM004137 for β1, NM181361 for β2, NM171829 for β3, and NM014505 for β4). We utilized β2 and β3 forms cloned in this lab [19], [20]. β4 was cloned in similar fashion. The β1 construct was originally obtained from O.B. McManus [13]. For mSlo3 eYFP insertion construct, we first introduced NheI and SphI sites at the linker between the two RCK domains (T645 and S646) of mSlo3, and then inserted eYFP PCR fragment with NheI and SphI sites, in-frame at the RCK linker region. The clone was identified by restriction enzymatic digestions and subsequently verified by DNA sequencing. The mSlo3 C-terminal in-frame attached eYFP was made from cloning of two PCR products, 93 bp SalI-NheI fragment from mSlo3 C-terminus and 730 bp NheI-KpnI fragment of eYFP, into SalI-KpnI digested pBF-mSlo3. A FLAG tag was introduced into both mSlo3 and mSlo1 via PCR using the primers containing the FLAG codon at the pBF vector for oocyte expression [21]. Similarly, a FLAG tag or a His tag (containing 7 histidines) was introduced into mSlo3 or β1–β4, respectively, first at the pBF vector and then transferred to pFastBac1 vector (Invitrogen) for Sf9 cells infection.

### Electrophysiology

All currents were recorded in the inside-out configuration with an Axopatch 200 amplifier (Molecular Devices, Sunnyvale, CA). The Clampex program from the pClamp software package (Molecular Devices) was used for data acquisition control. Procedures for reliably recording Slo3 current have been described [22], but typically utilize pipettes with less than 1 MΩ resistance. Pipette tips were coated with Sylgard (Sylgard 184, Dow Chemical Corp.) and then fire-polished. Gigaohm seals were formed in frog Ringer (in mM, 115 NaCl, 2.5 KCl, 1.8 CaCl_2_, 10 HEPES, pH 7.4). Excised patches were moved into flowing test solutions bathing the cytosolic membrane face. The standard pipette/extracellular solution was (in mM) 140 K-methanesulfonate, 20 KOH, 10 HEPES, 2 MgCl_2_, pH 7.0. The composition of solutions bathing the cytoplasmic face of the patch membrane contained (in mM) 140 K-methanesulfonate, 20 KOH, 10 HEPES, and 5 EGTA with the pH adjusted to values from 6.0 to 8.5 with HCl or KOH. Solutions were exchanged at the cytosolic face of membranes by a local application system in which a perfusion pipette (100–200 µ tip) was packed with six PE10 tubes [10].

All experiments were at room temperature (∼22–25°C). Salts for solution preparation were obtained from Sigma (St. Louis, MO).

### Data Analysis

Analysis of current recordings was accomplished either with Clampfit (Molecular Devices) or with programs written in this laboratory. For Slo3 currents, G/V curves were generally constructed from measurements of steady-state current, because the fast tail current compromises determination of tail current conductances [22]. For families of G/V curves obtained within a patch, conductances were normalized to estimates of maximal conductance obtained at pH 8.5. Individual G/V curves were fit with a Boltzmann function of the following form: 
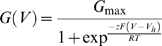
(1)where G_max_ is the fitted value for maximal conductance at a given pH, V_h_ is the voltage of half maximal activation of conductance, and *z* reflects the net charge moved across the membrane during the transition from the closed to the open state.

Analysis of current variance as a function of mean current (σ^2^/I) followed procedures utilized previously for Slo3 [22]. To better constrain estimates of N and Po, σ^2^/I relationships obtained under multiple conditions in the same patch were fit simultaneously [23].

### GFP detection

Mice were deeply anesthetized by ketamine-xylazine solution, perfused with 0.01 M PBS (in mM, 150 NaCl, 3 KCl, 8 Na_2_HPO4, 2 KH_2_PO4, pH 7.4), followed by 4% Paraformaldehyde (PFA, SIGMA) via the left ventricle. Testes were dissected and postfixed in 4% PFA fixative buffer for 3 hours at 4°C, cryoprotected sequentially with 20% and 30% sucrose, embedded in O.C.T. (Optimum Cutting Temperature) compound (Tissue-Tek) and frozen on dry ice. Testes sections were cut at 25 µ in a cryostat (Leica), placed on glass slides and mounted with Gel Mount Aqueous Mounting Medium (Sigma). Mouse cauda epididymal sperm or blood cell suspensions in PBS were dropped on glass slides, covered with coverslips, and mounted with nail oil. GFP signals were captured with an Olympus IX81 confocal microscope (Olympus).

### Mouse sperm and Sf9 cell membrane protein preparation

Mouse sperm were isolated from the cauda epididymis of 10 mature males and thoroughly washed in PBS. Sperm were suspended in 3 ml PBS (containing 10 µl Protease Inhibitor Cocktail and 10 µl 1.5 M PMSF) and sonicated 4 times at the setting of 3 by a Branson Sonifier 150 for 15 seconds in 4°C cold room. The pellet was discarded after centrifugation at 4,000 rpm for 10 minutes. The supernatant was ultra-speed centrifuged in a 4°C Ti70 rotor at 38,000 rpm for 1 hour. The membrane pellet was resuspended in 0.5 ml lysis buffer (50 mM Na phosphate, 150 mM NaCl, 10 mM KCl, 1% Triton X-100, 0.5% Sodium Deoxycholate, 0.1% SDS, pH 7.2) with Kontes Pellet Pestle Cordless Motor (Fisher Scientific). The membrane suspension was placed on ice for 1 hour, followed by centrifugation at 12,000 rpm for 10 minutes. The supernatant was saved as the sperm membrane protein preparation in the −80°C freezer. For preparation of Sf9 cell membrane protein, 100 ml Sf9 cell culture (1.5–2.0×10^6^ cells/ml, infected by recombinant Baculovirus for 72 hours) was collected by centrifugation at 4,000 rpm for 10 minutes. Then, cell pellets were resuspended in 20 ml PBS and broken in a nitrogen bomb by allowing equilibration at 500–1000 psi for 15 minutes on ice. Subsequently, Sf9 cell membrane proteins were then prepared as described above for sperm membrane proteins.

### Immunoprecipitation

Membrane proteins were prepared from Sf9 cells infected with mSlo alone or together with hβ subunits. 4 ml membrane protein preparation was mixed with 4 ml lysis buffer (containing 2.5% Triton X-100). As a negative control, a membrane protein preparation from Sf9 cells infected with mSlo α subunits alone was mixed with hβ membrane protein preparation obtained from separately infected Sf9 cells. Immunoprecipitation was performed with the FLAG Immunoprecipitation Kit (Sigma). After adding 100 µl Anti-FLAG M2 affinity gel to the membrane protein mixture and rocking samples in a 4°C cold room for 16 hours, the affinity gel was collected by centrifugation at 12,000 rpm for 20 seconds. The gel pellet was washed thrice with 1 ml ice-cold 1% Triton X-100 in PBS. FLAG-tagged protein complexes were eluted with 80 µl 3XFLAG Peptide (Sigma) solution (0.225 mg/ml in lysis buffer) and stored in the −80°C freezer.

### Western blot analysis

Samples typically containing 5–10 µg total protein with a total volume of 10 µl were mixed well with 10 µl SDS loading buffer containing 100 mM DTT, placed at room temperature for 30 minutes before loading to 4–20% or 8% Precise Protein Gels (Pierce). Protein markers applied were Bench Mark Pre-stained Protein Ladder (Invitrogen, USA). Proteins were transferred to Immobilon-P PVDF (Millipore) membranes with the Trans-Blot Semi-Dry Transfer System (Bio-Rad). Membranes were blocked with 5% nonfat milk in Tris-buffered saline-Tween 20 (TBST) buffer at room temperature for 1 hour, followed by overnight incubation at 4°C in 2% milk containing monoclonal anti-mSlo3 N2/16 antibody (1∶100 for sperm membrane proteins; 1∶2000 for Sf9 membrane proteins and immunoprecipitation products, Antibodies Inc.), monoclonal anti-mSlo1 L6/60 antibody (1∶2000, Antibodies Inc.) or polyclonal anti-6His antibody (1∶2000, ab9108, Abcam Inc.). After being washed thrice with TBST for 15 minutes, membranes were incubated either with Mouse Trueblot Ultra HRP-conjugated anti-mouse IgG (1∶1000, eBioscience), or with stabilized HRP-conjugated Goat anti-mouse or Goat anti-rabbit IgG (10 µg/ml, 1∶1000, Pierce) in 2% milk at room temperature for 1 hour. HRP-labeling was developed using Amersham ECL Plus Western Blotting Detection System (GE Healthcare).

### Detection of total and cell surface expression of Slo3 and β4 in oocytes

Oocyte surface proteins were labeled with biotin following established procedures [24]. 50 biotinylated oocytes were homogenized in 1 ml lysis buffer by seven passages through a 20 gauge needle and three passages through a 27 gauge needle, with 10 µl Protease Inhibitor cocktail (Sigma) added to protect proteins from digestion. Samples were centrifuged at 2,000 rpm for 10 minutes at 4°C to remove the yolk. 0.2 ml 10% Triton X-100 was added to the collected supernatant, followed by rocking at 4°C for 30 minutes to solubilize membrane proteins. After centrifugation at 12,000 rpm for 10 minutes, 100 µl of the supernatant was collected and supplemented with 100 µl SDS buffer. This sample was used to detect total β4 subunit expression. Separately, 500 µl of the supernatant was supplemented either with 60 µl of previously washed anti-FLAG M2 agarose (Sigma) to be used as the total FLAG-tagged Slo3 expression sample, or with 60 µl Streptavidin agarose (Prozyme) to prepare Slo3 and β4 surface expression samples. After overnight incubation in a 4°C cold room, agarose beads were collected and washed thrice with 1 ml lysis buffer (containing 1% Triton X-100). FLAG-tagged proteins were eluted from the anti-FLAG M2 agarose beads by incubation in 60 µl 3XFLAG solution (0.225 mg/ml 3XFLAG peptide in lysis buffer) on ice for 30 minutes. Biotin labeled proteins were eluted from the streptavidin agarose beads with 75 µl SDS loading buffer. Regular Western blotting procedures were applied to the samples, with monoclonal anti-mSlo3 antibody (1∶200, Antibodies Inc) or monoclonal anti-mβ4 antibody (1∶200, Antibodies Inc) as the primary antibody and the HRP-conjugated goat anti-mouse IgG antibody (1∶1000, Pierce) as the secondary antibody, correspondingly. Integrated density of the immunoblot band in the X-ray film was quantified using ImageJ 1.40 g software (Rasband, W.S., ImageJ, U. S. National Institutes of Health, Bethesda, Maryland, USA, http://rsb.info.nih.gov/ij/, 1997–2008).

### RNA extraction and quantitative RT-PCR

Total RNA from mouse testes or cauda epididymal sperm was isolated using the RNeasy Plus Mini Kit (Qiagen, Valencia, CA) following the manufacturer's recommendations. Before the reverse transcription, total RNA was treated to remove genome DNA with the DNA-free Kit (AM1906, Applied Biosystems). cDNA was synthesized using the Retroscript Kit (AM1710, Applied Biosystems). For the negative control groups, all components except the reverse transcriptase MMLV-RT were included in the reaction mixtures. Real-Time PCR with specific primers (Table S1) was performed using Power SYBR Green PCR Master Mix (Applied Biosystems). Mouse β-actin gene was utilized here as the homogenous standard. The running protocol extended to 40 cycles consisting of 95°C for 15 s and 60°C for 1 minute using an Applied Biosystems 7500 Fast Real-time PCR system. PCR specificity was checked by dissociation curve analysis and DNA electrophoresis.

### Protein Deglycosylation

Membrane proteins from virus-infected Sf9 cells were treated with N-Glycanase PNGase F (Prozyme) under denaturing conditions. 35 µl membrane proteins were mixed with 10 µl 5×Reaction Buffer (Prozyme) and 2.5 µl Denaturation Solution (Prozyme). Prior to adding the enzyme, samples were heated at 100°C for 5 minutes and then cooled to room temperature. 2.5 µl Detergent Solution (Prozyme) and 2 µl PNGase F (2.5 U/ml) were added to the reaction mixture. Samples were incubated at 37°C water bath for 3 hours to remove N-glycosylated sugars. Because significant aggregation of hβ2 occurred after 100°C heating, a modified protocol was applied on hβ2 samples: the heating treatment was omitted and the incubation time with enzyme was increased to 24 hours.

## Results

### Coexpression of human or mouse KCNMB4 with mouse Slo3 increases Slo3 current and surface membrane expression in *Xenopus* oocytes

**Figure 1:**
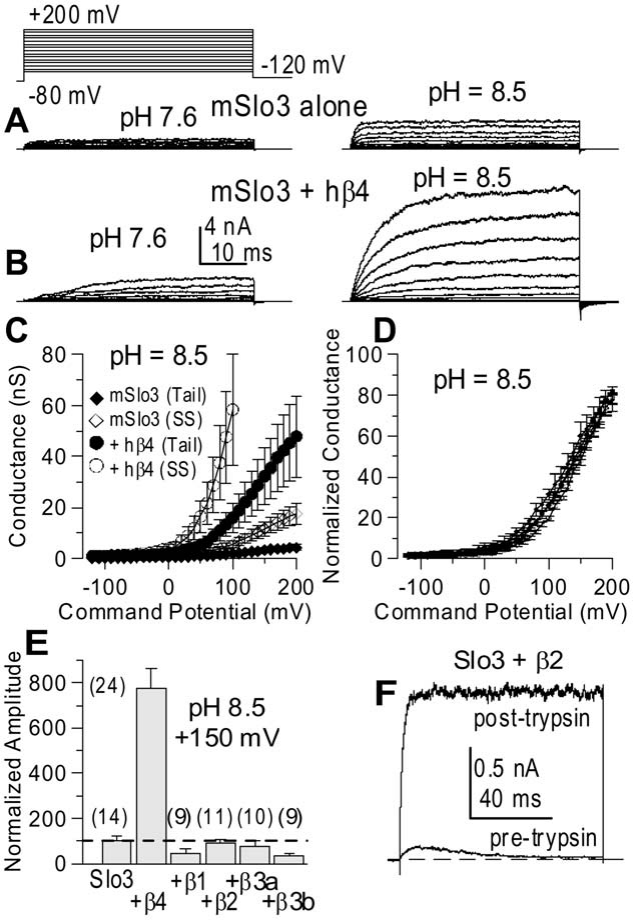
KCNMB4 increases Slo3 macroscopic conductance in *Xenopus* oocytes over 8-fold. Recordings were done in inside-out patches 5 days after cRNA injection. In A–D, an identical amount of mSlo3 cRNA was injected into oocytes all from the same batch. In A, mSlo3 currents were activated with the indicated voltage-protocol either at pH 7.6 (left) or pH 8.5 (right). In B, currents resulting from coexpression of mSlo3 and hβ4 were recorded as in A. In C, mean steady-state and tail conductances measured as a function of voltage at pH 8.5 were determined for both Slo3 (n = 9) and Slo3+hβ4 (n = 8). Steady state current for Slo3+hβ4 at voltages over +100 mV typically exceeded the range of the recording system. In D, GV curves were normalized to the maximum conductance estimated from a fit of a Boltzmann function (Eq. 1) to each curve. Numbers of patches for Slo3 steady-state current and Slo3+hβ4 tail current are the same as in C. Slo3 tail GV curves were obtained from 5 patches with current amplitudes sufficient to allow meaningful Boltzmann fitting, while Slo3+hβ4 steady state GV curves were from 4 patches with current amplitudes at +200 mV within the range of the recording system. In E and F, all patches were from a separate batch of oocytes injected with identical amounts of Slo3 cRNA and maintained for 5 days at 17°C. In E, steady-state current amplitudes measured at pH 8.5 and +150 mV are compared for Slo3 alone and for coexpression with other BK β subunits. In F, Slo3 +β2 current measured at pH 8.5 and +150 mV was robustly increased after trypsin application. A pre-pulse at −160 mV for 100 ms was used to drive channels to resting states before activation.

Figure 1A and 1B show representative currents obtained from inside-out patches five days after injection for Slo3 expressed either without or with hβ4 (mSlo3 cRNA: 10 ng/oocyte; +/−hβ4 cRNA: ∼20 ng/oocyte). At any pH, Slo3+β4 currents were much larger than currents for Slo3 alone (Fig. 1C). On average, the net Slo3 conductance per patch was about 8-fold larger in the presence of hβ4 subunit (Fig. 1C and E). However, the presence of hβ4 did not change the normalized G-V relationship, suggesting that the increased current observed with hβ4 does not result from a shift of voltage-dependent channel gating (Fig. 1D). We also examined currents resulting from Slo3 coexpressed with other BK β subunits. Although for β2 there was trypsin-sensitive inactivation indicating coassembly of Slo3 with β2 (Fig. 1F), the ability to increase Slo3 currents was unique to the β4 subunit (Fig. 1E). In contrast, coexpression of hβ4 with Slo1 α did not increase average current density. In patches excised from oocytes four days following cRNA injection, with 10 µM cytosolic Ca^2+^ at +150 mV, Slo1+hβ4 currents were 65.4±22.3% (n = 9) of those obtained with Slo1 alone (n = 10).

**Figure 2:**
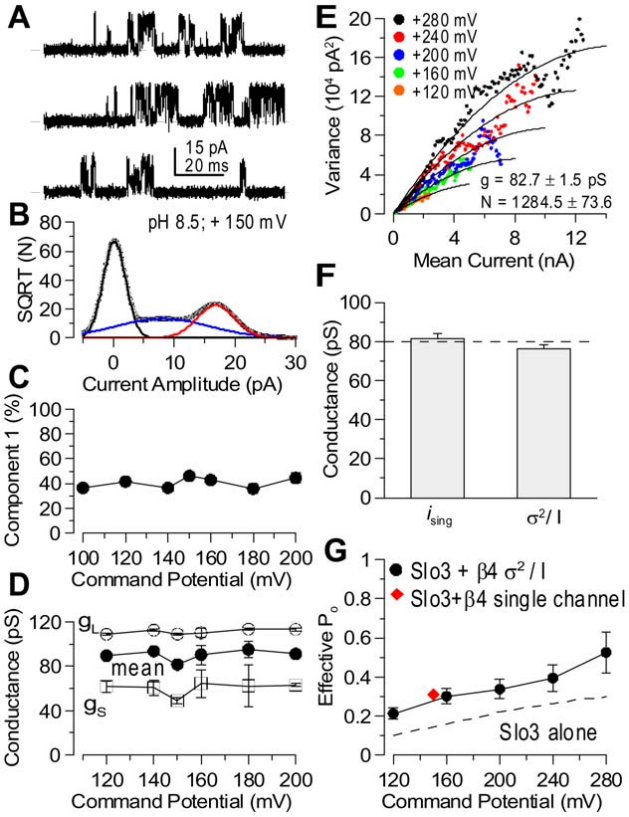
Coexpression of Slo3 with β4 does not appreciably increase single channel conductance or effective Po. In A, traces show openings from a patch with a single Slo3+β4 channel at +150 mV with pH 8.5 (filtering at 10 kHz). In B, a total amplitude histogram of all digitized current values from a 995 ms record of Slo3+β4 single channel activity is plotted, showing a substantial number of current values between the closed level (0 pA) and the largest open level (∼18 pA). Histograms were fit with a 3-component Gaussian function to estimate the fraction and amplitude of the closed level, the smaller current level (g_S_) and the larger current level (g_L_). In C, the g_S_ fraction of all open current levels is plotted as a function of voltage (3–5 patches). In D, the mean conductance for g_S_, g_L_, and the overall mean is plotted as a function of voltage. In E, σ^2^ is plotted as a function of mean current level for current recorded from a single patch at 5 different voltages. For each voltage, a set of 100 repeated steps to the nominal voltage was used to activate currents from which σ^2^ and mean current, I, were determined for each time point in the average. The σ^2^/I relationships at multiple voltages were fit simultaneously by σ^2^(I,V) = g*V*I - I^2^/N with best fit values indicated by the solid lines, yielding g = 82.7±1.5 pS and N = 1284.5±73.6 channels. In F, the mean conductance estimated from single channel measurements at +150 mV (n = 5) and the single channel conductance estimated from σ^2^/I estimates (n = 6) are compared, along with previous estimates for Slo3 alone (dotted line). In G, estimates of Po for Slo3+β4 patches at pH 8.5 obtained either from σ^2^/I estimates (black circles, n = 3–6) or from the mean single channel current estimate (red diamond, n = 5) are plotted as a function of voltage and compared to previous estimates from Slo3 (dotted line).

An increase of macroscopic current amplitude may result from either an increase in channel number, an enhancement of open probability, an increase of single channel conductance, or a combination of these factors. At the single channel level, Slo3 channels exhibit flickery openings which result in a total amplitude histogram requiring at least two Gaussian components to obtain a reasonable fit of the open levels [25]. Slo3+hβ4 single channels also exhibit similar openings (Fig. 2A) and two Gaussian components were required to fit the open levels in the total amplitude histogram obtained from a single Slo3+hβ4 channel (Fig. 2B). The estimated mean conductance for Slo3+hβ4 channels was essentially identical to that of single Slo3 channels (Fig. 2C and D; [25]). Thus, the hβ4-mediated increase in macroscopic Slo3 current does not arise from an effect on single channel conductance.

Another characteristic of Slo3 channel is that, even at +300 mV, the limiting open probability is only about 0.3 [22], [25]. To assess whether the increase in current amplitude in the presence of β4 might arise from changes in limiting Po, we measured the current variance at different mean current levels. The σ^2^/I relationships at different voltages were fit to estimate the single channel conductance (g) and N, the number of channels in a patch (Fig. 2E). The estimated average single channel conductance was similar to that obtained from single channel analysis (Fig. 2F). Furthermore, the σ^2^/I relationship for Slo3+β4 currents shows little hint of a parabolic behavior that would imply that Po values over 0.5 are obtained. From the fitted values of N and g, an estimate of effective Po at a particular voltage was obtained from Po(V) = I_max_/(N*g*V), with I_max_ the maximum current amplitude recorded at that voltage. The estimates of Po at positive potentials for Slo3+β4 (Fig. 2G) are perhaps somewhat higher than those observed for Slo3 alone but within the previously observed range [22]. Thus, the increase in Slo3 current resulting from coexpression with β4 must arise from an increase in total channel number, and not an effect on single channel Po or single channel conductance.

**Figure 3:**
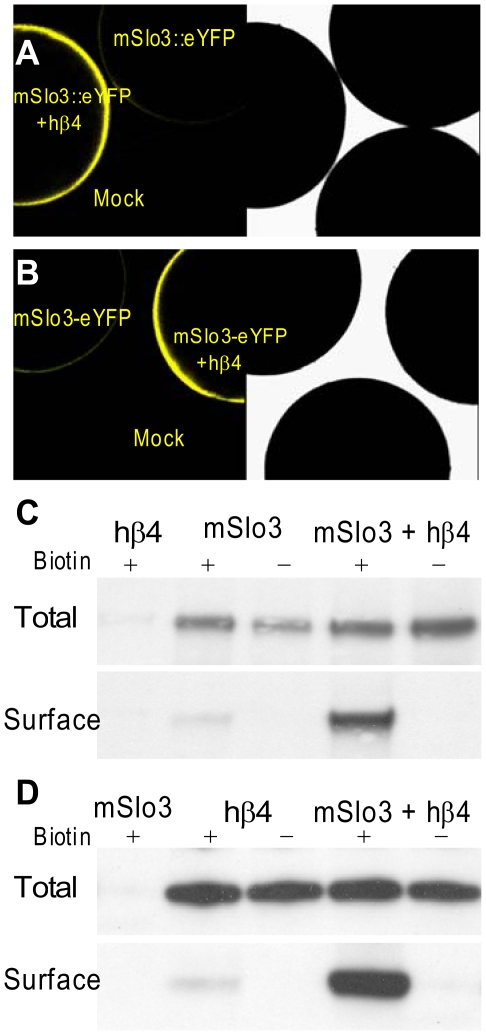
β4 increases surface expression of Slo3. In A, oocytes from the same batch were injected either with mSlo3::eYFP (eYFP inserted between RCK domains) alone, mSlo3::eYFP+hβ4, or with H_2_O. Confocal sections revealed weak fluoresence in oocytes injected only with mSlo3 alone, while hβ4 strongly increased surface fluoresence. For illustrative purposes, an oocyte of each category was positioned together and viewed either with fluorescent (left) or normal illumination (right). In B, oocytes were injected with Slo3-eYFP (eYFP attached to C-terminus) with or without hβ4. As in A, hβ4 substantially increased surface fluorescence. In C and D, Slo3 was FLAG tagged. In C, Slo3 either in samples of total protein or surface protein expressed either alone or together with β4 in *Xenopus* oocytes was detected with Western blotting. Total Slo3 protein was purified by immunoprecipitation with anti-FLAG M2 agarose. After “blotting” of the immunoprecipitated proteins, 1∶200 anti-Slo3 antibody was used as the primary antibody. Integrated density of the immunoblots was quantified using ImageJ 1.40 g software (http://rsb/info.nih/gov/ij/). For total protein samples, the amount of Slo3 protein in the presence of β4 is 1.2-fold of that in the absence of β4. The corresponding ratio for surface protein is 12. In D, total and surface expression of β4+/−Slo3 are compared. Monoclonal anti-mβ4 antibody (1∶200) served as the primary antibody. The ratio of β4 amount in the presence of Slo3 compared to that without Slo3 is 1 or 11, for total protein or surface protein samples, respectively.

To confirm that the total number of Slo3 subunits in the surface membrane is increased by the hβ4 subunits, an eYFP-inserted Slo3 (Fig. 3A) or eYFP-attached Slo3 (Fig. 3B) was expressed with or without hβ4. In matched pairs of oocytes, the hβ4 subunit increased Slo3-eYFP fluorescence signal dramatically. A mouse β4 subunit also produced a similar increase in Slo3 surface membrane fluorescence increase and Slo3 current amplitude enhancement.

Is the surface expression enhancement of Slo3 caused by coassembly with hβ4 a consequence of an increase in total Slo3 expression? To address this, we used biotin labeling of surface protein to compare both total protein and surface protein of Slo3 expressed with or without hβ4 subunits in frog oocytes. The surface level of Slo3 was increased about 11-fold by coexpression with hβ4 subunits (Fig. 3C, Surface), which is consistent with our YFP-tagged Slo3 results (Fig. 3A and B). In contrast, the total protein level of Slo3 was only slightly changed by hβ4 (Fig. 3C, Total). These results imply that hβ4 increases the surface expression of Slo3 by affecting Slo3 trafficking rather than translation of Slo3. Interestingly, we found that the surface expression of hβ4 was also markedly increased by coexpression with Slo3 (Fig. 3D, Surface), although Slo3 showed little effect on total hβ4 (Fig. 3D, Total). The mechanisms underlying this reciprocal enhancement of Slo3 and β4 on surface expression require further investigation.

Coexpression with hβ4 also altered Slo3 activation kinetics. Time constants for activation and deactivation were determined and plotted as a function of command potential (Fig. S1), showing that β4 slowed both the activation and deactivation time course. These functional changes further support the idea that β4 coassembles with Slo3. However, because of the lack of functional change when Slo3 is coexpressed with either β1, β3a or β3b, the coassembly of these subunits with Slo3 is uncertain. To provide some biochemical tests for the ability of different β subunits to coassemble with Slo3, we utilized FLAG-tagged Slo3 and His-tagged β subunits in the insect Sf9 expression system (Fig. S2). For all Slo3/β subunit combinations, following immunoprecipitation with an anti-FLAG antibody, subsequent development of the Western blots with an anti-His antibody clearly revealed specific β subunit bands (Fig. S2C). In contrast, mixing of membranes from Sf9 cells separately infected with FLAG-Slo3 or a His-β subunit did not allow coimmunoprecipitation (Fig. S2D). These results indicate that the KCNMB family of auxiliary subunits are competent to coassemble with the Slo3 subunit, although they provide no information about the specificity or strength of these interactions.

### β4 and Slo3 may co-exist in mouse spermatogonia

**Figure 4:**
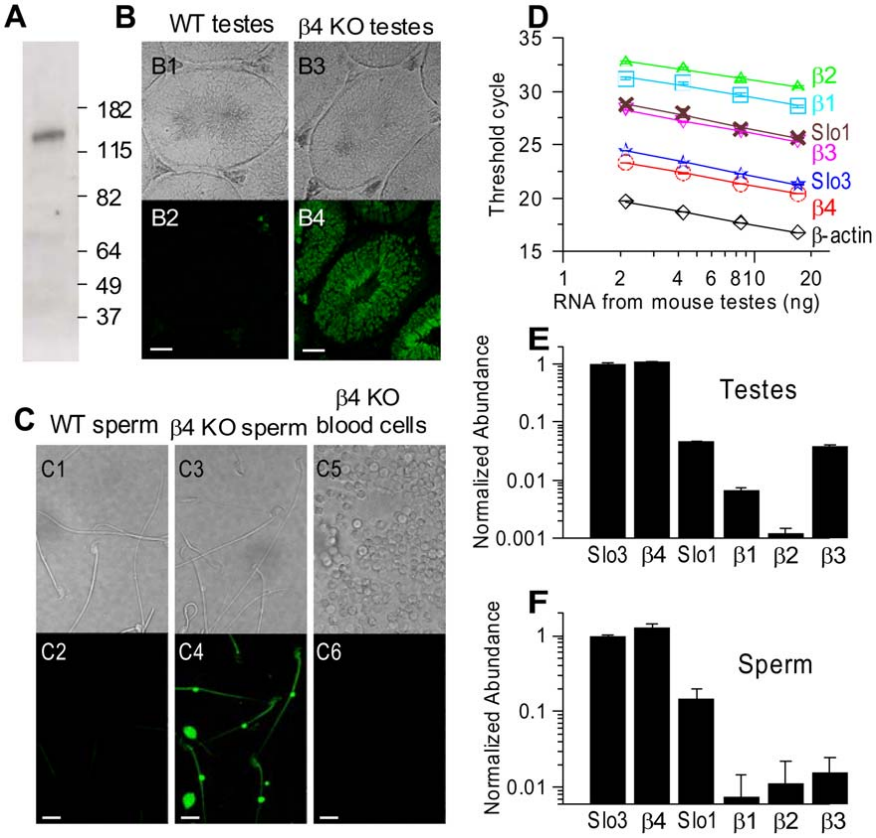
β4 may specifically co-exist with Slo3 in mouse sperm. In A, membrane proteins from WT mouse cauda epididymal sperm were detected with anti-mSlo3 antibody as primary antibody. In B, a section from wild-type mouse testis showing well-defined seminiferous tubules is shown with normal (B1) and fluorescent (B2) illumination. Background fluorescence is limited to spaces between seminiferous tubules. A section of testis from a β4 KO mouse is shown under normal (B3) and fluorescent (B4) illumination revealing prevalent GFP fluorescence within seminiferous tubules. In C, similar comparisons are shown for sperm collected from caudal epididymis of both WT (C1, C2) and β4 KO mice (C3, C4). No significant fluorescence was detected from blood cells of β4 KO mice (C5, C6). In D, RNA - threshold cycle relationship reveals that the abundance of β4 and Slo3 mRNA in testes is similar. Each data point is an average from three experiments. The solid lines are linear fits with the slope values as follows: β-actin, −3.26; Slo1, −3.62; Slo3, −3.51; β1, −2.94; β2, −2.62; β3, −3.34; β4, −3.27. In E (testes, n = 3) and F (sperm, n = 3), the relative abundance of each tested subunit compared to Slo3 is displayed. Abundance was calculated from 2^−dCt^, with dCt = Ct(subunit) - Ct(Slo3). Ct(subunit) stands for the threshold cycle number of the corresponding subunit, and Ct(Slo3) is the average threshold cycle number for Slo3 obtained from three parallel experiments for either testes or sperm. Scale bars: 50 µm in B, 10 µm in C.

Based on the association of β4 subunits with Slo3 in oocytes, we hypothesize that β4 subunits may be regulatory components of Slo3 channels if expressed together in normal cells. Since previous RT-PCR, Northern analysis and in situ hybridization showed that Slo3 is primarily expressed in spermatocytes [5], we examined whether β4 may be present together with Slo3 in spermatocytes. We first confirmed, using Western blots on membrane proteins prepared from mouse cauda epididymal sperm, that a clear band with size similar to the predicted Slo3 molecular weight could be detected, supporting the presence of Slo3 in mouse sperm (Fig. 4A). However, with available antibodies, we were unable to reveal a specific β4 band in similar membrane preparations. Taking advantage of the availability of β4 KO mice, in which the deleted exon was replaced by an eGFP tag [26], we examined eGFP expression in spermatocytes from β4 KO mice. The resulting eGFP is a soluble protein which would be expected to be found throughout the cytosol of any cell in which the β4 promoter is active. Sections of testes from both WT and KO mice were observed under fluorescent microscopy. Compared to the weak background fluorescence restricted to spaces between seminiferous tubules from WT sections (Fig. 4B1 and B2), sections from KO mice showed substantial eGFP fluorescence within seminiferous tubules (Fig. 4B3 and B4). This indicates the β4 promoter is active in spermatocytes from β4 KO mice. Furthermore, in comparisons of sperm collected from caudal epididymis of WT and KO mice, while fluorescence was minimal in sperm from WT mice (Fig. 4C1 and C2), sperm from β4 KO mice consistently exhibited clear eGFP fluorescence (Fig. 4C3 and C4). No significant fluorescence was detected in blood cells of KO mice, indicating the expression of eGFP in sperm is specific rather than a universal consequence of the deletion of β4 exon (Fig. 4C5 and C6). These results suggest that the β4 gene is active in sperm, thereby allowing the possibility that β4 subunits are available for coassembly with sperm Slo3 subunits.

To determine the relative abundance of β4 and Slo3 message in mouse testes and sperm, we employed quantitative RT-PCR. mRNA levels of Slo1 and other β subunits were also determined. The ubiquitously expressed β-actin was utilized as a control. Threshold cycle numbers (Ct) were employed to compare the relative mRNA amount of individual subunits [27]. RNA extracted from testes was serially diluted to give dose-response curves, which displayed comparable slope values for each subunit, indicating that the method had similar detection efficiency among the subunits tested (Fig. 4D). β4 and Slo3 in testes exhibited nearly equivalent mRNA abundance, at a level much higher than that of other BK subunits (Fig. 4D). This result was confirmed by RT-PCR on mRNA extracted from cauda epididymal sperm (Table S2). The abundance of each subunit message relative to Slo3 message was determined for both testes (Fig. 4E) and sperm (Fig. 4F).

### Slo3 coassembles with β4 in the presence of Slo1

**Figure 5:**
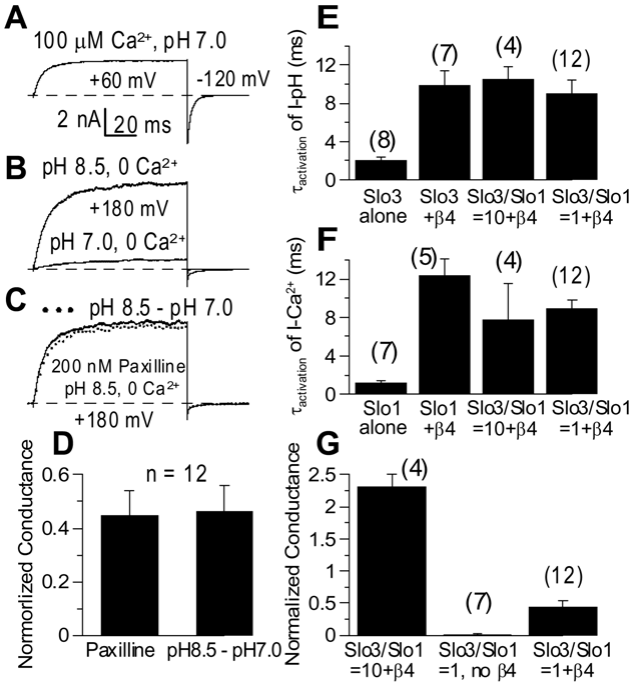
β4 regulates Slo3 even in the presence of Slo1. In A–D, currents were obtained from oocytes injected with Slo3 and Slo1 at 1∶1 ratio, together with hβ4 (Slo3/Slo1 = 1+hβ4). Example traces in A–C were recorded from the same patch in the order from A to C. Tail currents were recorded at −120 mV for all cases. In A, the trace shows exclusively Ca^2+^-dependent current. In B, currents were recorded with 0 Ca^2+^ at +180 mV either at pH 7.0 or pH 8.5 to reveal the pH-dependent current. In C, current was recorded at +180 mV under 0 Ca^2+^ at pH 8.5 with 200 nM paxilline applied to block Slo1 channels. The dotted line indicates the pH-dependent current revealed by subtraction of current recorded at pH 7.0 from that recorded at pH 8.5 in B. In D, the amplitude of the paxilline-resistant current is compared to that of the pH-dependent current. Both paxilline-resistant conductance and pH-activated conductance were normalized to that of the Ca^2+^-dependent current from the same patch. In E, the activation time constants at +180 mV of the pH-dependent currents obtained from different Slo3/Slo1/β4 combinations are compared. In F, the activation time constants at +60 mV of the Ca^2+^-dependent currents obtained from different Slo3/Slo1/β4 combinations are compared. In G, the pH-dependent conductance relative to the Ca^2+^-dependent conductance in each combination is shown.

Although the Slo1 mRNA level was much less than that of Slo3 and β4 in sperm (Fig. 4D–F), Slo1 subunits might compete with Slo3 for β4 subunits perhaps occluding a potential regulatory effect of β4 on Slo3. To address whether β4 maintains its ability to modify Slo3 channels in the presence of Slo1, we examined currents in oocytes injected with different Slo1/Slo3/β4 mRNA combinations. For the simple case in which Slo1 and Slo3 do not coassemble, two components of current, Ca^2+^-dependent and pH-dependent, would be expected. We took the current recorded with 100 µM Ca^2+^ (pH 7.0) at +60 mV as Ca^2+^-dependent current (Fig. 5A). Under these recording conditions, the open probability of Slo1 (and Slo1+β4) channels almost reached saturation [8], [18], whereas the open probability of Slo3 (and Slo3+β4) was very low [22], [25]. On the other hand, pH-dependent current was obtained with pH at 8.5 (0 Ca^2+^) at +180 mV, after applying 200 nM paxilline to the patch for at least two minutes to completely block Slo1 channels (Fig. 5B and C; [28], [29]). Slo3 channels are completely resistant to paxilline at this concentration (unpublished data). One concern is whether Slo1 and Slo3 may form heteromultimeric channels under these experimental conditions. Two arguments support the view that functional heteromultimeric channels do not contribute to any of the observed currents. First, the paxilline-resistant current showed a nonlinear instantaneous rectification behavior similar to that of Slo3 (or Slo3+β4) current (Fig. 5C), while the Ca^2+^-dependent current exhibited linear instantaneous current behavior similar to Slo1 (or Slo1+β4) current (Fig. 5A). Second, the paxilline-resistant component of current was essentially identical in amplitude to the component of current activated by pH 8.5 (Fig. 5B–D). This strongly suggests that heteromultimeric formation of Slo3 and Slo1 into functional channels is not significant.

When Slo1 was injected together with Slo3 and β4 at mRNA levels one tenth of that of Slo3 (mimicking the mRNA ratio of Slo3 to Slo1 in sperm (Fig. 4D–F)), the pH-dependent current exhibited slowed activation time constants characteristic of those observed for Slo3+β4 current (Fig. 5E). In fact, even with 1∶1 Slo3/Slo1 injection ratio, the pH-dependent current still showed prolonged activation time constants (Fig. 5E), indicative that Slo3 subunits are associated with β4 subunits even under this highly competitive situation. On the other hand, the Ca^2+^-dependent current at both 1∶1 and 10∶1 Slo3/Slo1 injection ratios showed activation constants slower than that of Slo1 alone current (Fig. 5F), consistent with the idea that the Ca^2+^-dependent current also involves associated β4 subunits [18]. These results suggest that there is no strong preference for β4 to associate with Slo1 over Slo3.

To obtain information about the relative density of these two current components in different mRNA combinations, the pH-dependent conductance in each patch was normalized to the Ca^2+^-dependent conductance of the same patch. When Slo3 and Slo1 were injected at a ratio of 10∶1, the pH-dependent conductance greatly exceeded the Ca^2+^-dependent conductance in the presence of β4 (Fig. 5G). These results imply that, under conditions, in which Slo1 mRNA exists at a level much less abundant than Slo3 and β4 mRNA, the conductance arising from Slo3 channels greatly exceeds the conductance arising from Slo1 channels. With Slo1 injected at a level similar to that of Slo3, the majority of the conductance was Ca^2+^-dependent in the absence of β4 (Fig. 5G). In contrast, when β4 was presented, the pH-dependent conductance was increased to about 45% of the Ca^2+^-dependent conductance (Fig. 5G), reflecting the surface expression enhancement effect of β4 on Slo3 channels. Considering the lower open probability (∼0.3 under these recording conditions) and smaller single channel conductance of Slo3 channels compared to Slo1 channels [22], [25], the actual membrane density of Slo3 channels is expected to be greater than that of Slo1 channels. This also suggests that Slo3 subunits will associate with β4 subunits even in the presence of comparable amounts of Slo1 subunits.

### Mouse reproductive capacity is not affected by deletion of the β4 gene

To test whether β4 might influence the function of sperm, we compared the reproductive capacity of wild type and β4 KO mice. Individual male mice, either β4 KO (n = 10, 8-weeks old) or wild type (n = 10, 8-weeks old), were each housed with two wild type female mice (C57BL/6J, 9-weeks old) per cage. Since experienced mothers have less tendency to eat their pups, only the number of pups from the second litter of each female mouse was compared. For the wild type group, 130 pups were recorded from 17 litters, giving an average of 7.6±0.5 pups per litter. Correspondingly, each litter from the β4 KO group included an average of 7.2±0.7 pups, with 94 pups collected from 13 litters. Although a difference in total litter number was observed between the two groups, other factors rather than sperm function may account for this difference (two β4 KO males failed to fertilize either of two females, while all WT males produced pups). The fact that average litter size of both groups was identical implies that the reproductive capacity of mice is not markedly affected by the deletion of the β4 gene.

## Discussion

The results described here have two key implications. First, an auxiliary subunit of the KCNMB family, β4, is a candidate regulatory partner of the pH-dependent Slo3 K^+^ channel in mouse sperm. Second, KCNMB auxiliary subunits may have functional roles independent of Ca^2+^- and voltage-activated BK channels.

Prevailing dogma suggests that KCNMB subunits are only involved in formation of BK channels [30]. In fact, the β4 subunit has been considered the primary auxiliary subunit involved in assembly of many brain BK channels [11], [31], [32] and KO of the β4 subunit is associated with alterations in neuronal excitability [26]. Here, we establish that all members of the KCNMB family are capable of coassembly with the Slo3 α subunit in heterologous expression systems. We also establish that β4 confers functional effects on Slo3 currents including enhancement of surface expression and alteration of gating kinetics. Furthermore, the results provide strong support for the idea that Slo3 and β4 are coexpressed in native mouse spermatocytes, raising the possibility that β4 influences Slo3 function in sperm. While the much higher abundance of β4 mRNA compared to other β subunits implies that the presence of β4 in sperm is relatively specific, the significantly lower abundance of Slo1 mRNA relative to β4 and Slo3 strengthens our hypothesis that the β4 subunit may play roles not associated with Slo1 channels. Similarly, in other tissues it may be possible that other members of the KCNMB family may also have functions unrelated to BK channels.

The conclusion that β4 increases the surface expression of Slo3 was supported by electrophysiological analysis, confocal microscopy with eYFP tagged Slo3 constructs, and detection of Slo3 surface membrane protein by biotin labelling. Because there is little change of Slo3 total protein expression in the presence of β4, it is likely that β4 increases Slo3 surface expression by affecting Slo3 trafficking. Interestingly, we found that the surface expression of β4 is also markedly enhanced by Slo3. The reciprocal alteration of trafficking could arise from a number of mechanisms. First, Slo3 or β4 may each contain a retention signal that is masked by association with each other. Second, coassembly of β4 with Slo3 may increase the membrane insertion effectiveness of Slo3 channel. As a consequence, the membrane insertion of β4 is also increased by association with Slo3. Third, the Slo3+β4 protein complex may be less subject to degradation processes resulting in a slower turnover rate compared to Slo3 alone or β4 alone protein. Whatever the mechanism that results in increased surface expression of both β4 and Slo3, these effects appear to be specific for β4 in comparison to other KCNMB subunits.

Slo3 is the primary candidate for the gene responsible for a pH-regulated KSper current in mouse spermatocytes [4]. Although the present results reveal that β4 may be a potential Slo3 interaction partner in sperm, the reported effects of β4 on Slo3 do not help resolve the differences between KSper and Slo3 expressed in oocytes [4], [22], [25]. Specifically, the β4 subunit does not change the voltage-dependence of Slo3 current significantly (Fig. 1D), which is stronger than that of KSper [4]. Therefore, for Slo3 to mediate KSper current in spermatocytes, other regulatory factors in addition to β4 may be required to explain the differences between KSper and Slo3.

## Supporting Information

Table S1 Primers used for Real-Time RT-PCR
(0.03 MB DOC)

Table S2 Average threshold cycle values for Slo1, Slo3 and KCNMB family β subunits in mouse testes and cauda epididymal sperm. RT: Reverse Transcriptase
(0.03 MB DOC)

Figure S1 Kinetic properties of Slo3+β4 channels differ from Slo3 channels.In A, the normalized activation time course for currents either with mSlo3 alone, or with coexpression of mSlo3+hβ4 are compared at +200 mV and pH 8.5. In B, mean activation time constants for Slo3 alone (8 patches) and Slo3+hβ4 (4–8 patches) are compared as a function of activation voltage at pH 8.5. In C, normalized tail current time course for Slo3 with and without hβ4 are compared at pH 8.5 and −120 mV. hβ4 resulted in consistently slower tail currents, with two exponential components required to fit Slo3 alone and three required to fit Slo3+hβ4. The two faster components match those required to fit Slo3, while a slower third component was observed in the presence of β4.
(0.03 MB PDF)

Figure S2 Expression and coassembly of β4 subunits with Slo3 in Sf9 cells. Slo3 was FLAG-tagged and all tested β subunits were tagged with 7 Histidines (His-tagged). In A, B & C, 1∶2000 anti-6His antibody was used as the primary antibody to detect β subunits. In A, β subunits in total membrane proteins from Sf9 cells infected with β subunits alone or together with mSlo3 were detected with Western blotting. Columns are: a, mSlo3; b, mSlo3+hβ1; c, hβ1; d, mSlo3+hβ2; e, hβ2; f, mSlo3+hβ3b; g, hβ3b; h, mSlo3+hβ4; i, hβ4. In B, larger molecular weight bands were removed by treatment with N-Glycanase PNGaseF, indicating that the occurrence of multiple bands is a consequence of N-glycosylation at different levels. The predicted molecular weight of deglycosylated His-tagged β subunits is: hβ1-7His, 22.7 kD; hβ2-7His, 28.1 kD; hβ3b-7His, 30.0 kD; hβ4-7His, 24.9 kD. In C, β subunits in IP products pulled down by anti-FLAG M2 agarose (Slo3 was FLAG-tagged) were detected by Western blotting. Membrane protein preparations were obtained either from Sf9 cells co-infected with mSlo3 and a β subunit or from mixtures of membranes from cells infected with mSlo3 or β subunits separately. Columns correspond to: 1, mSlo3+hβ1; 2, mix mSlo3 with hβ1; 3, mSlo3+hβ2; 4, mix mSlo3 with hβ2; 5, mSlo3+hβ3b; 6, mix mSlo3 with hβ3b; 7, mSlo3+hβ4; 8, mix mSlo3 with hβ4. In D, Slo3 from the same IP products as in panel C was detected with 1∶2000 anti-mSlo3 antibody. Column order is identical to panel C.
(0.03 MB PDF)
