# Supplementary material for: Interactions between β Subunits of the KCNMB Family and Slo3: β4 Selectively Modulates Slo3 Expression and Function
Source: PLoS One. 2009 Jul 3;4(7):e6135. doi: 10.1371/journal.pone.0006135 (PMC2701609; doi:10.1371/journal.pone.0006135)
Supplement: Table S1 — Primers used for Real-Time RT-PCR (0.03 MB DOC) [file pone.0006135.s001.doc]

**Table S1. Primers used for Real-Time RT-PCR**

| gene | primer | amplicon length |
| --- | --- | --- |
| mβ1 | Mm_Kcnmb1_1_SG QuantiTect Primer Assay from Qiagen(cat# QT00101500) | 128 bp |
| mβ2 | Mm_Kcnmb2_1_SG QuantiTect Primer Assay from Qiagen(cat# QT00128695) | 86 bp |
| mβ3 | Mm_Kcnmb3_1_SG QuantiTect Primer Assay from Qiagen(cat# QT00323708) | 127 bp |
| mβ4 | Mm_Kcnmb4_1_SG QuantiTect Primer Assay from Qiagen(cat# QT00155988) | 86 bp |
| mSlo1 | Forward: 5’-TCTCAGCATTGGTGCCCTCGTAAT-3’  Reverse: 5’-GTAGAGGAGGAAGAACACGTTGAA-3’ | 127 bp |
| mSlo3 | Forward: 5’-CTTTGTACTAAGCATCGGGTCTCT-3’  Reverse: 5’-GAAGCTAAAGAAAGCGTTGAAACT-3’ | 127 bp |
| β-actin | Forward: 5’-TGGAGAAGAGCTATGAGCTGCCTG-3’  Reverse: 5’-GTAGTTTCATGGATGCCACAGGAT-3’ | 127 bp |
