# Supplementary material for: Interactions between β Subunits of the KCNMB Family and Slo3: β4 Selectively Modulates Slo3 Expression and Function
Source: PLoS One. 2009 Jul 3;4(7):e6135. doi: 10.1371/journal.pone.0006135 (PMC2701609; doi:10.1371/journal.pone.0006135)
Supplement: Table S2 — Average threshold cycle values for Slo1, Slo3 and KCNMB family β subunits in mouse testes and cauda epididymal sperm. RT: Reverse Transcriptase (0.03 MB DOC) [file pone.0006135.s002.doc]

Table S2. Average threshold cycle values for Slo1, Slo3 and KCNMB family β subunits in mouse testes and cauda epididymal sperm. RT: Reverse Transcriptase

| Gene | Testes total RNA(8.5ng)  +RT | Testes total RNA(8.5ng)  -RT | Sperm total RNA(20ng)  +RT | Sperm total RNA(20ng)  -RT |
| --- | --- | --- | --- | --- |
| β-actin (n=3) | 18.21±0.01 | 36.33±0.45 | 23.35±0.03 | 36.74±0.93 |
| mSlo1 (n=3) | 26.26±0.01 | undetected | 32.20±0.51 | undetected |
| mSlo3 (n=3) | 21.82±0.05 | undetected | 29.28±0.03 | undetected |
| mβ1 (n=3) | 29.02±0.13 | undetected | undetected | undetected |
| mβ2 (n=3) | 31.57±0.38 | undetected | undetected | undetected |
| mβ3 (n=3) | 26.489±0.03 | undetected | 34.72±0.36 | undetected |
| mβ4 (n=3) | 21.68±0.02 | 38.57±1.13 | 28.94±0.17 | 37.83±1.15 |
