# Supplementary figures and images for: Interactions between β Subunits of the KCNMB Family and Slo3: β4 Selectively Modulates Slo3 Expression and Function
Source: PLoS One. 2009 Jul 3;4(7):e6135. doi: 10.1371/journal.pone.0006135 (PMC2701609; doi:10.1371/journal.pone.0006135)

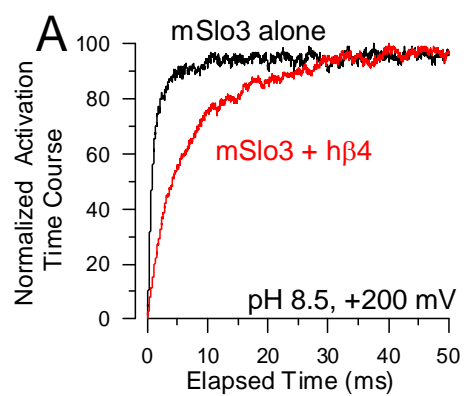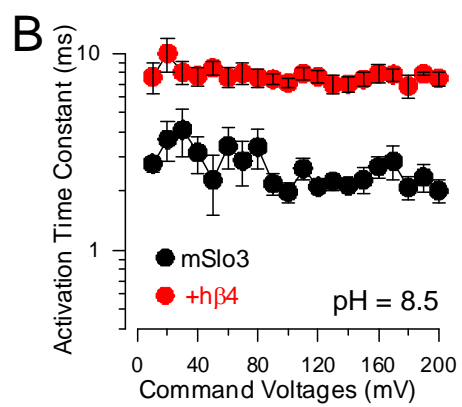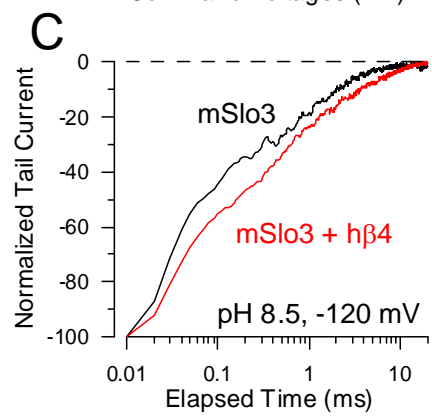

Supplement: Figure S1 — Kinetic properties of Slo3+β4 channels differ from Slo3 channels.In A, the normalized activation time course for currents either with mSlo3 alone, or with coexpression of mSlo3+hβ4 are compared at +200 mV and pH 8.5. In B, mean activation time constants for Slo3 alone (8 patches) and Slo3+hβ4 (4–8 patches) are compared as a function of activation voltage at pH 8.5. In C, normalized tail current time course for Slo3 with and without hβ4 are compared at pH 8.5 and −120 mV. hβ4 resulted in consistently slower tail currents, with two exponential components required to fit Slo3 alone and three required to fit Slo3+hβ4. The two faster components match those required to fit Slo3, while a slower third component was observed in the presence of β4. (0.03 MB PDF) [file pone.0006135.s003.pdf]

**A**

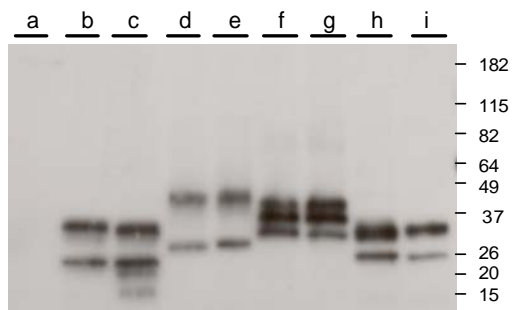

**C**

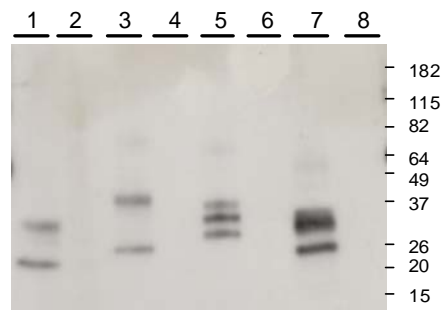

**B**

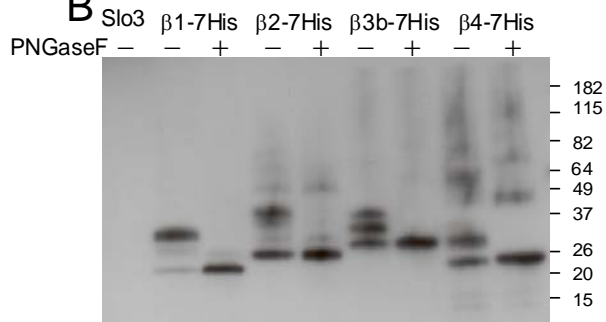

**D**

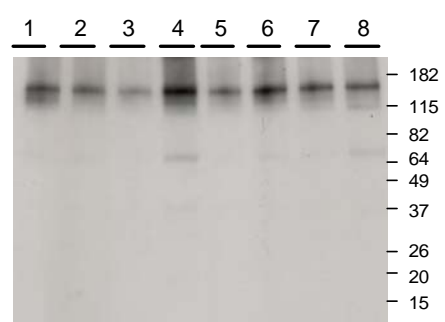

Supplement: Figure S2 — Expression and coassembly of β4 subunits with Slo3 in Sf9 cells. Slo3 was FLAG-tagged and all tested β subunits were tagged with 7 Histidines (His-tagged). In A, B & C, 1∶2000 anti-6His antibody was used as the primary antibody to detect β subunits. In A, β subunits in total membrane proteins from Sf9 cells infected with β subunits alone or together with mSlo3 were detected with Western blotting. Columns are: a, mSlo3; b, mSlo3+hβ1; c, hβ1; d, mSlo3+hβ2; e, hβ2; f, mSlo3+hβ3b; g, hβ3b; h, mSlo3+hβ4; i, hβ4. In B, larger molecular weight bands were removed by treatment with N-Glycanase PNGaseF, indicating that the occurrence of multiple bands is a consequence of N-glycosylation at different levels. The predicted molecular weight of deglycosylated His-tagged β subunits is: hβ1-7His, 22.7 kD; hβ2-7His, 28.1 kD; hβ3b-7His, 30.0 kD; hβ4-7His, 24.9 kD. In C, β subunits in IP products pulled down by anti-FLAG M2 agarose (Slo3 was FLAG-tagged) were detected by Western blotting. Membrane protein preparations were obtained either from Sf9 cells co-infected with mSlo3 and a β subunit or from mixtures of membranes from cells infected with mSlo3 or β subunits separately. Columns correspond to: 1, mSlo3+hβ1; 2, mix mSlo3 with hβ1; 3, mSlo3+hβ2; 4, mix mSlo3 with hβ2; 5, mSlo3+hβ3b; 6, mix mSlo3 with hβ3b; 7, mSlo3+hβ4; 8, mix mSlo3 with hβ4. In D, Slo3 from the same IP products as in panel C was detected with 1∶2000 anti-mSlo3 antibody. Column order is identical to panel C. (0.03 MB PDF) [file pone.0006135.s004.pdf]
